# Integrated design of an aerial soft-continuum manipulator for predictive maintenance

**DOI:** 10.3389/frobt.2022.980800

**Published:** 2022-09-20

**Authors:** Xinrui Yang, Mouad Kahouadji, Othman Lakhal, Rochdi Merzouki

**Affiliations:** Laboratory CRIStAL, University of Lille, Lille, France

**Keywords:** aerial soft manipulator, 3D printing in construction, deep learning, crack detection, neural network

## Abstract

This article presents an integrated concept of an aerial robot used for predictive maintenance in the construction sector. The latter can be remotely controlled, allowing the localization of cracks on wall surfaces and the adaptive deposit of the material for *in situ* repairs. The use of an aerial robot is motivated by fast intervention, allowing time and cost minimizing of overhead repairs without the need for scaffolding. It is composed of a flying mobile platform positioned in stationary mode to guide a soft continuum arm that allows to reach the area of cracks with different access points. Indeed, some constructions have complex geometries that present problems for access using rigid mechanical arms. The aerial robot uses visual sensors to automatically identify and localize cracks in walls, based on deep learning convolutional neural networks. A centerline representing the structural feature of the crack is computed. The soft continuum manipulator is used to guide the continuous deposit of the putty material to fill the microscopic crack. For this purpose, an inverse kinematic model-based control of the soft arm is developed, allowing to estimate the length of the bending tubes. The latter are then used as inputs for a neural network to predict the desired input pressure to bend the actuated soft tubes. A set of experiments was carried out on cracks located on flat and oblique surfaces, to evaluate the actual performances of the predictive maintenance mechatronic robot.

## 1 Introduction

The construction domain faces many constraints; one of them is the lifespan of the structures which depends on the type of materials used in these structures. The used materials (concrete, metal, wood, etc.) are subject to external conditions such as climate changes and humidity. These latter cause a lot of damage and degradation that can produce cracks and it may lead to a breakdown of the building. Repairing these cracks at the first degradation stage is a subject of predictive maintenance. It can be less costly, with less mobilization time and safety for workers on site. Therefore, this topic is recent original research in designing and controlling autonomous robots for crack repair to anticipate important structure degradation.

Concerning concrete surfaces, it exists and works in relation to the identification and characterization of cracks and other structural damages. Aggelis et al. (2011) worked on a robust methodology for damage evaluation in concrete before it becomes visible. Also, Albishi et al. (2012) worked on metal surfaces and proposed a new sensor based on complementary split-ring resonators to detect surface cracks.

In addition, the research area for automation of predictive maintenance has become wider in these last years because of the electronic and robotic revolution. There exist various concepts and works related to the use of robots for crack repair. In this topic, Zhu et al. (2019) presented an arm system for roadway crack sealing, and Tsai et al. (2013) developed an automatic method using a mobile robot for crack detection and sealing for roadways.

The most proposed concepts of predictive maintenance are based on vision systems. The image processing technology is the main step for structural health monitoring tasks, including crack detection, quality, and safety for both modern and old constructions. For a crack detection task, various approaches which did not require human involvement were proposed. Based on whether the detection system has contact with a wall surface, the methods can be divided into two main categories: based on contact and based on vision (without contact). Contact-based methods usually use embedded sensors to detect and characterize mechanical fractures based on the data recorded from the sensors. Palermo et al. (2020) proposed a customized design of an integrated tactile and proximity sensor. Lau (2003) used an optical fiber to assess concrete structures. Vision-based methods (Yao et al., 2019) rely on image structure features and they detect the surface defects by analyzing the texture, skeleton, edge (Canny, for example), and spectrum of the image.

Recently, deep learning convolutional neural networks (CNNs) have been used in image-related tasks such as target tracking (Feng et al., 2019) and semantic segmentation (Li et al., 2019). In addition to the fast improvement of computational ability, a CNN also has a strong ability in feature extraction, which is a key point in this kind of detection task. He et al. (2017) proposed a region-based convolutional neural network architecture for instance segmentation. Mohan and Poobal (2018) and Silva and Lucena (2018) studied and reviewed multiple studies in the field of deep learning based on crack detection and compared the performances in different experimental conditions.

Researchers have validated the feasibility and satisfying performance of deep learning CNN approaches for surface defect detection. Dorafshan et al. (2018a) investigated the feasibility of using a CNN based on an AlexNet dataset (Krizhevsky et al., 2017), in the inspection of concrete buildings using small unmanned aerial vehicles. Dorafshan et al. (2017) and Dorafshan and Maguire (2018) proved the feasibility and challenges of fatigue crack detection in bridge structures as well. As for the comparative study, the CNN shows very robust performance compared to the traditional, well-known edge detection methods. Dorafshan et al. (2018b) compared the performance of common edge detectors and deep CNNs for image-based crack detection in concrete structures. It shows the advantage of CNNs to perform crack detection, compared to edge detection algorithms which are more influenced by external noises. In addition to crack detection on concrete wall surfaces, deep CNNs can also be used in the detection of other categories of surface imperfections, with rather good performance. Perez et al. (2019), proposed a CNN to detect the dampness of buildings, while discussing the cause and effects of this type of defect.

With the development and massive implementation of deep learning convolutional neural networks in defect detection tasks, we focus, in this work, on developing a model based on a CNN to identify cracks on concrete surfaces. The transfer learning method is used in the development of a crack detection model, i.e., we fine-tune the hyperparameters of the crack detection model based on a pre-trained model for multi-class instance segmentation. When a crack is detected and located, it is then classified in the following classes: microscopic (width < 5 mm), mesoscopic (5 mm < width < 10 mm), and macroscopic (width > 10 mm). In the following work, we were interested in microscopic cracks. Then, their shapes and structural features can be computed, extracted, and sent to the crack reparation mechatronic robot to perform the predictive maintenance process.

A soft-continuum manipulator has been introduced these last years for inspection activities in the construction domain. The monitoring of the surface state can be automated to detect any appearance of cracks. Several systems have been developed to automate the inspection process in some specific environments, such as subway tunnels (Zhang et al., 2014), flexible pavement surfaces (Oliveira and Correia, 2013) (Shi et al., 2016), bridge decks (Prasanna et al., 2016), and buildings (Yan et al., 2017) (Chen et al., 2016). De Paz et al. (2013) used a hexapod robot, named Hex-piderix, with a stereo camera to inspect the cracking on surfaces such as walls or roofs of buildings. Access to these places is sometimes difficult for the operators. Some used unmanned aerial vehicle (UAV) systems to diagnose cracks on surfaces of the buildings. Crack detection is essential for health monitoring of built infrastructure. An integrated system that enables revisiting crack locations during building inspections by means of a quadrature UAV is presented by Kucuksubasi and Sorguc (2018). Phung et al. (2017) used a UAV the data collection to create a 3D model of the targeted structure by using laser scanners. For kinematic modeling of a soft continuum arm, data-driven approaches have been considered to improve the performance model reconstruction, such as by Karlik and Aydin (2000) and Daachi et al. (2012), where neural networks were used to approximate the behavior of a large number of Degrees of Freedom (DoF) robot manipulators, allowing to develop adaptive controllers in the presence of nonlinearities. A hybrid controller combining both the model-based and neural network approaches has been proposed by Jiang et al. (2017) and Reinhart et al. (2017). Jiang et al. (2017) presented a control algorithm for a manipulator that can be extended to several segments in a 2D plane. Its kinematics is solved in two levels. In the first level, an analytical model based on the gradient descent method is used to determine the optimized positions of all segment peaks based on predefined measurements. The second level uses neural networks to determine the pressures of each segment, taking into account the viscoelasticity property. Reinhart et al. (2017) proposed a hybrid, data-driven analytical approach for the early control of a bionic manipulator. The effectiveness of this approach was demonstrated by a reduction in tracking errors, which represents less than half of the errors observed when using the analytical model. Lakhal et al. (2015) used a multi-layer neural network to approximate the solutions of the inverse kinematic equations of the soft continuum robot, where the time-allocation for the learning process was considerable, due to the complex kinematic of the robot. A data-driven approach has also been used to approximate the soft arm kinematics by MELINGUI et al. (2014), to develop a kinematic controller (Melingui et al., 2015). A hybrid approach is introduced by Lakhal et al. (2015) for real-time solving of the soft arm model. It consists of a real-time resolution and implementation in case of trajectory tracking. A methodology of kinematic modeling and synthesis of the approximate solutions is proposed for the case of a continuum manipulator. The modeling approach is issued from a modeling of a series of parallel rigid manipulators, where the resolution of the nonlinear model is approximated using a neural network technique.

### 1.1 Study contribution

Repair of wall surfaces can require time and high precision. The automation of this operation allows for reducing the intervention time and keeping a good repair quality. Currently, the literature does not report on integrated solutions for all the following steps for crack repair, namely, detection, localization, preparation, and repair. In this study, we propose an integrated concept for automatic detection, localization, and *in situ* repair of microscopic cracks with a local intervention on the surface. It uses cooperative behavior between an unmanned aerial vehicle (UAV) and a soft continuum arm to facilitate the task of predictive maintenance of cracks on flat or oblique surfaces. The advantage of using a UAV is that it can scan a large area on the wall surface and reach the microscopic crack location in less time, instead of using a scaffold. The UAV control can be autonomous or with the operator in the loop. This last option is considered in this development, to consider the safety requirement of using the UAV in a confined space. Then, the soft arm allows guiding a nozzle to realize a continuous deposit of putty materials to fill the cracks. The choice comes from the fact that it can be operated on congested and narrow surfaces, due to its flexibility, after maintaining the UAV stable at its stationary position. This inherent flexibility makes them suitable for various applications, including tracking the shape of the cracks and operating in complex and congested environments. The use of continuum manipulators not only reduces the intervention time but they are also light and easy to install and transport. This work explains the steps of detection, localization, and repair of microscopic cracks in terms of local intervention with a continuous deposit of a putty material.

The article is organized as follows: Section 2 presents the predictive maintenance system for microscopic cracks in which the different components of the system are described. It also presents the deep learning approach for the identification and localization of cracks. The quantitative approach used to control the integrated mechatronic system is developed. The implementation and the obtained results are presented in section 3 followed by a discussion in section 4. The conclusion of the results and future work are provided in section 5.

## 2 Materials and methods

**FIGURE 1:**
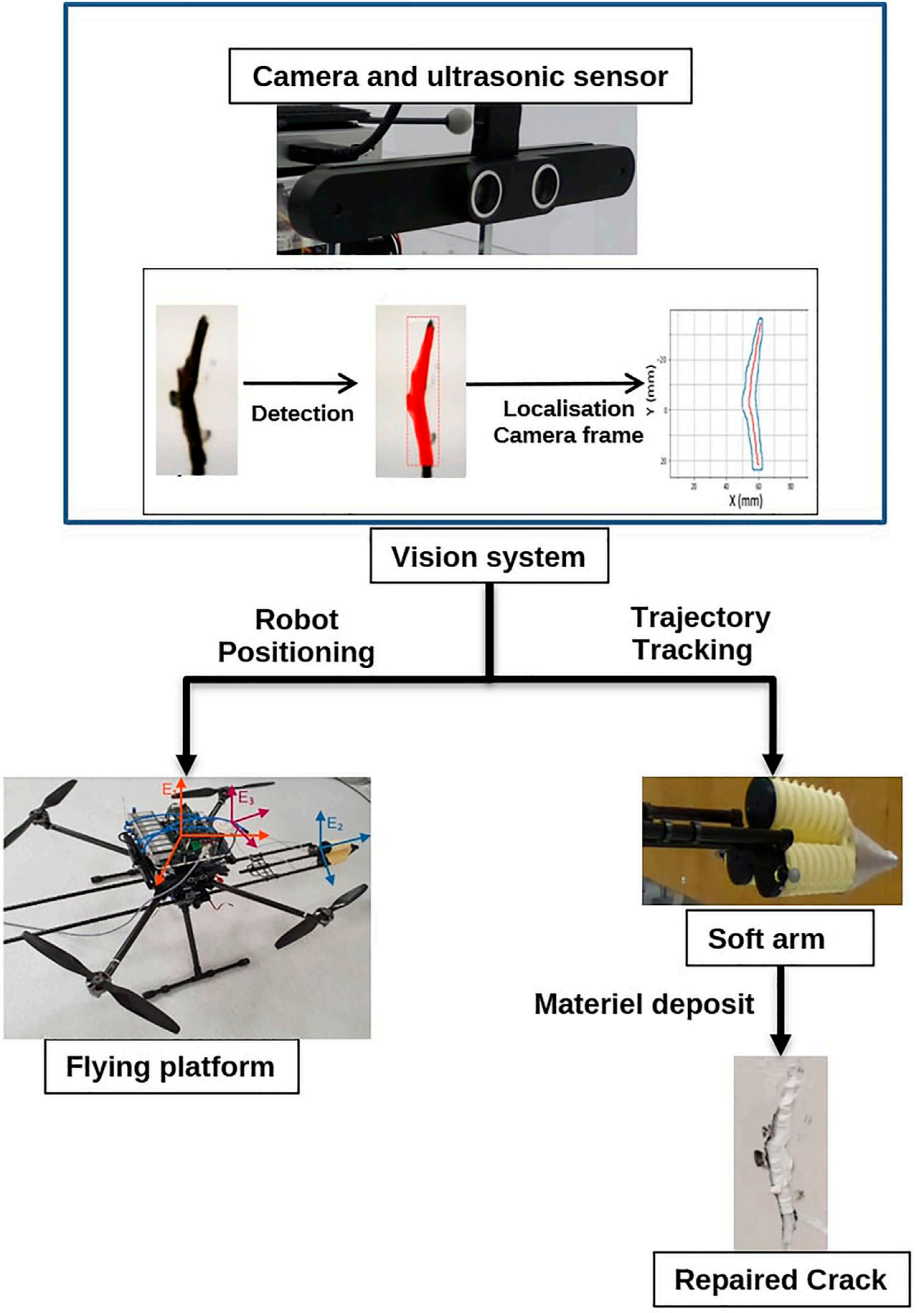
Principal of the predictive maintenance process.

The aim of our concept is to repair cracks automatically using robots and developed technologies. This task is achieved by performing several steps. The process of this operation called predictive maintenance is shown in Figure 1. The first step is to scan the structures with a camera embedded on the UAV and send the images and the current position of the system to an on-board computer to detect cracks. Once a crack is detected, according to the size of the crack and its location, the reparation decision will be taken and the system generates the shape of the crack and its centerline and they will be sent to the mobile platform and soft robot. The next step is to calculate the coordinates of this crack in the absolute frame of the UAV; this operation allows the mobile platform to move to the location of the crack. In this study, the movement of the mobile platform to the location of the crack is done manually using a joystick, and the UAV then keeps the same direction and altitude. Once the mobile platform is in a stationary position, the soft arm guides the nozzle along the centerline of the crack when it starts pumping the material. However, the accuracy of maintaining the UAV position can be affected by weather conditions, external contacts, GPS signal strength, etc.

### 2.1 System description

This part presents a general description of the predictive maintenance system, followed by a description of the methods developed for detecting and repairing microscopic cracks.

**FIGURE 2:**
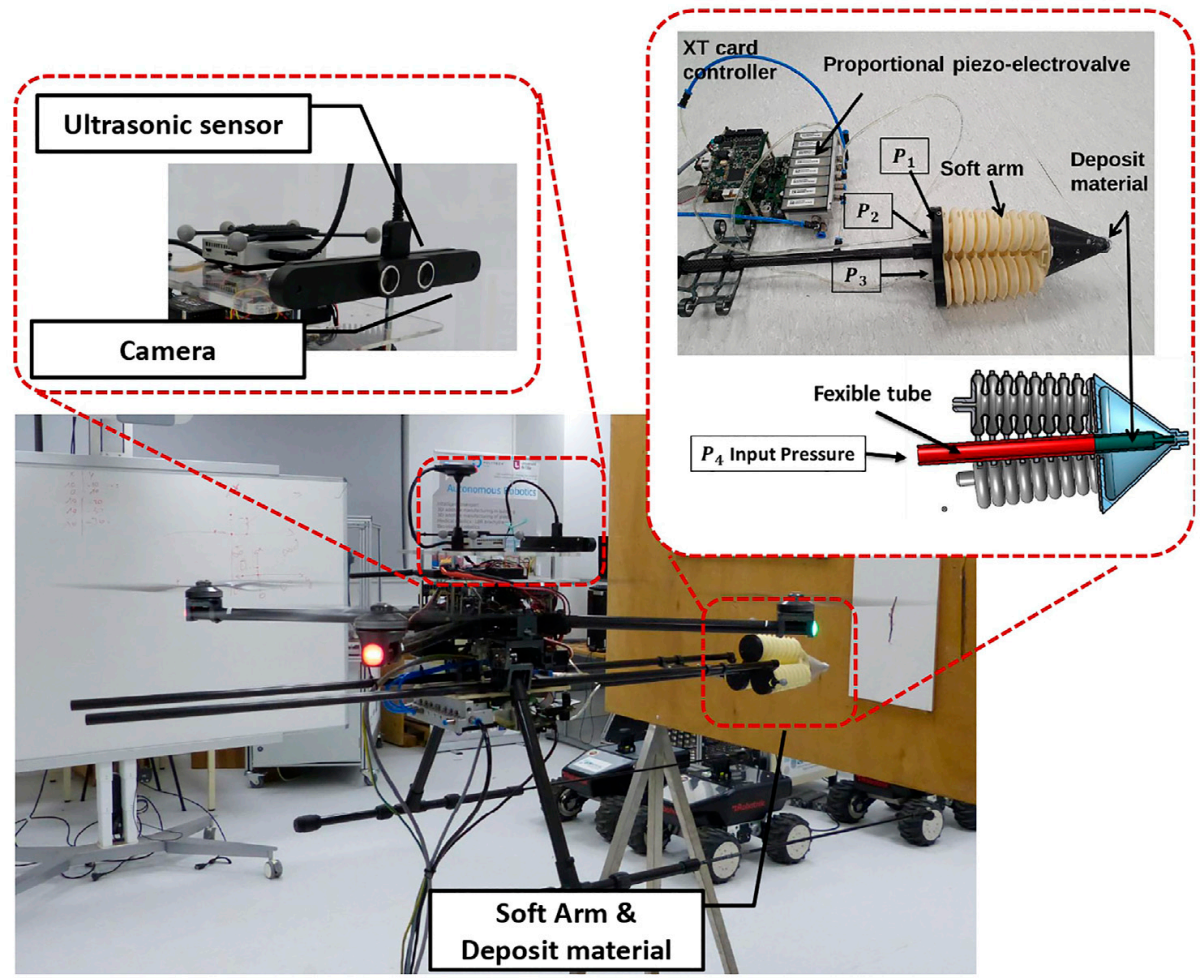
Predictive maintenance robot system.

The experimental platform is a prototype for predictive maintenance, composed of an unmanned aerial vehicle (UAV) and a soft arm, as shown in Figure 2. The idea is to design an industry-scale prototype that can automatically detect, locate, and repair microscopic cracks after filling putty materials from an embedded nozzle.

### 2.1.1 Soft arm

**FIGURE 3:**
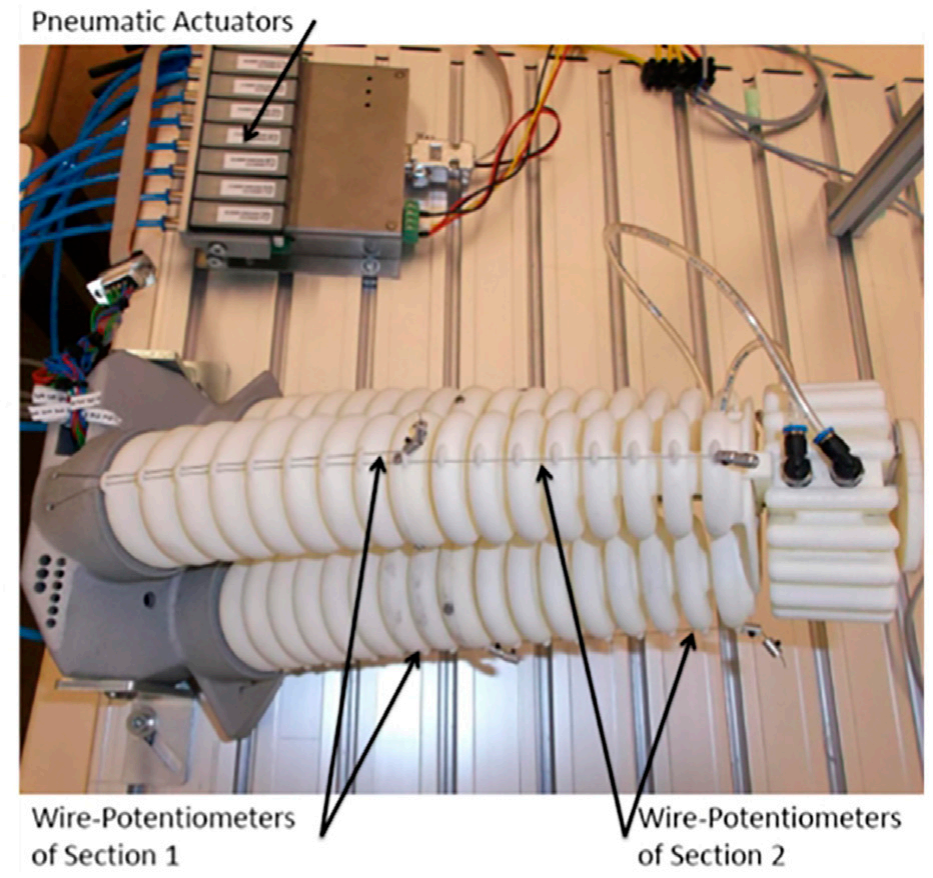
Two serial sections of the soft continuum manipulator.

The soft arm describes one section of the continuum manipulator called CBHA for compact bionic handling arm (Escande et al., 2015) as shown in Figure 3. It is a soft bionic manipulator inspired by an elephant trunk. It is a continuum robot and can be classified as a soft robot with intrinsic actuation. The rigidity of the soft arm is provided first by the polyamide material manufactured through a rapid prototyping process, and secondly, by the high number of interconnected bionic backbones, which provide it wide maneuverability compared to a rigid manipulator. Due to its flexible material, one section of the soft arm is lightweight (0.460 kg) and can carry out a maximal payload of 0.140 kg. From a macroscopic level, the bending section is composed of three continuous flexible backbone tubes (soft actuator). From a microscopic level, each tube is composed of bionic backbones. It is composed of three electro-pneumatic valves to control the pressure of each backbone tube. A total of three sensors are used to estimate the bending length from the three wire-potentiometers shown in Figure 3, which allow measuring the instantaneous lengths of the tube during bending. Also, reflective markers are installed on the tip and along the arm for shape control based on the Optitrack vision system shown in Figure 13.

### 2.1.2 Flying platform (unmanned aerial vehicle)

It is a four-rotor mobile flying platform equipped with four brushless DC motors. It is controlled by a Pixhawk autopilot, equipped with a GPS for outdoor missions and a PX4 flow sensor for indoor missions. Concerning indoor applications of the flying soft continuum manipulator technology, the miniature crack repair on interior surfaces of tall buildings and edifices of historical heritage is the main challenge. For that, a 3D scan of the indoor surfaces can be done *a priori* in offline, for the automatic detection and localization of the target cracks. The advantage of the optical flow is the position estimation using ultrasonic data and the image flow. For this considered UAV, the air supply unit and the power supply are placed on the ground. They are connected to the UAV through wires of 10 m. This is to reduce the overall weight of the system. The UAV is used as a guide platform for the soft arm. First, the UAV is stabilized in the stationary mode in front of the crack with the operator control in the loop. A wheeled contact (Figure 2) between the UAV and the wall has allowed to maintain the system stable. The parameters of the UAV are mass = 3.1 kg and size = 0.65 m. The UAV can carry out a load of 2 kg in the presented version. The soft arm, the nozzle system, and the piezo-electric valves have a weight of 1.25 kg. The fill of the crack is realized with the putty material traveling inside the nozzle. The Optitrack system is used to improve the stability of the UAV, where the tests are performed indoors, in absence of external disturbances. The current UAV system has been designed to hold a nozzle of 5 mm diameter, containing a maximum of 0.125 L of putty material (Figure 2). The nozzle is operated as a pre-loaded needle where the mandarin is replaced by a pressure-driven catheter through an electro-pneumatic valve shown in Figure 3. The vision guidance sensor of the UAV (Figure 2) collects image data through its embedded cameras and distance data through its ultrasonic sensors.

### 2.2 Crack detection and localization

This section describes a deep learning-based approach for the detection and localization of cracks. The approach explores the images captured by the embedded visual sensor (camera) of the UAV and detects existing cracks. Furthermore, a centerline (i.e., skeleton) of the crack is extracted to generate a tool path for the repairing task. Then the location of the crack within the frame of the drone and world frame is calculated. A deep learning instance segmentation model is adopted for the detection of cracks, followed by a morphological medial axis transform for the extraction of the centerline. The localization is realized by applying frame transformation to the calculated crack location.

### 2.2.1 Deep learning for crack detection

The detection of an existing crack on a wall surface is an essential step for structural health monitoring and for the procedure of predictive maintenance. Visual-based deep learning models are a set of widely implemented methods for this task. Some research works have implemented image classification (Flah et al., 2020) or object detection models (Park et al., 2020) for the detection and localization of cracks or cracked areas on concrete surfaces; however, those models do not meet our requirement for the purpose of automatic crack repair, because the tool path of the robot arm should overlap the structural shape of the crack. On the basis of the 2D image data available from visual sensors, we adopted an instance segmentation model, namely, Mask RCNN (He et al., 2017) for the detection and segmentation of cracks. Taking an image as input, the model is able to segment the crack area at the pixel level in the image frame.

Mask RCNN has been proven to be efficient and accurate in other single-target-single-class instance segmentation tasks in multiple fields including medical science (Anantharaman et al., 2018) and agriculture (Jia et al., 2020; Hameed et al., 2022).

An image dataset is prepared based on two public concrete crack image datasets containing over 100,000 images in total (Özgenel, 2017; Dorafshan et al., 2018c; Maguire et al., 2018). The images in those datasets are classified into two categories according to the existence of cracks, but without annotation. Since we need to train an instance segmentation model, instead of using directly the datasets which are ready for training a binary classification model, we select certain images to create an annotated crack image dataset. When selecting the images to annotate, different environmental conditions are considered, such as shadows and the complexity of the concrete background, to improve the performance of the model.

The annotated dataset contains RGB images of two different sizes (256 × 256 pixels and 227 × 227 pixels); each image contains between 0 and 4 cracks. With a computer vision annotation tool (CVAT), we have annotated 300 images containing 493 crack instances and have split them into train (260 images) and validation (40 images) sets. For each image, a binary mask is created, in which the value 0 is assigned to background pixels and the value *i* is assigned to the pixels of the *i*-th crack. To enhance the diversity of the dataset, data augmentation methods are applied randomly, such as image flipping, blur, and shadowing.

**FIGURE 4:**
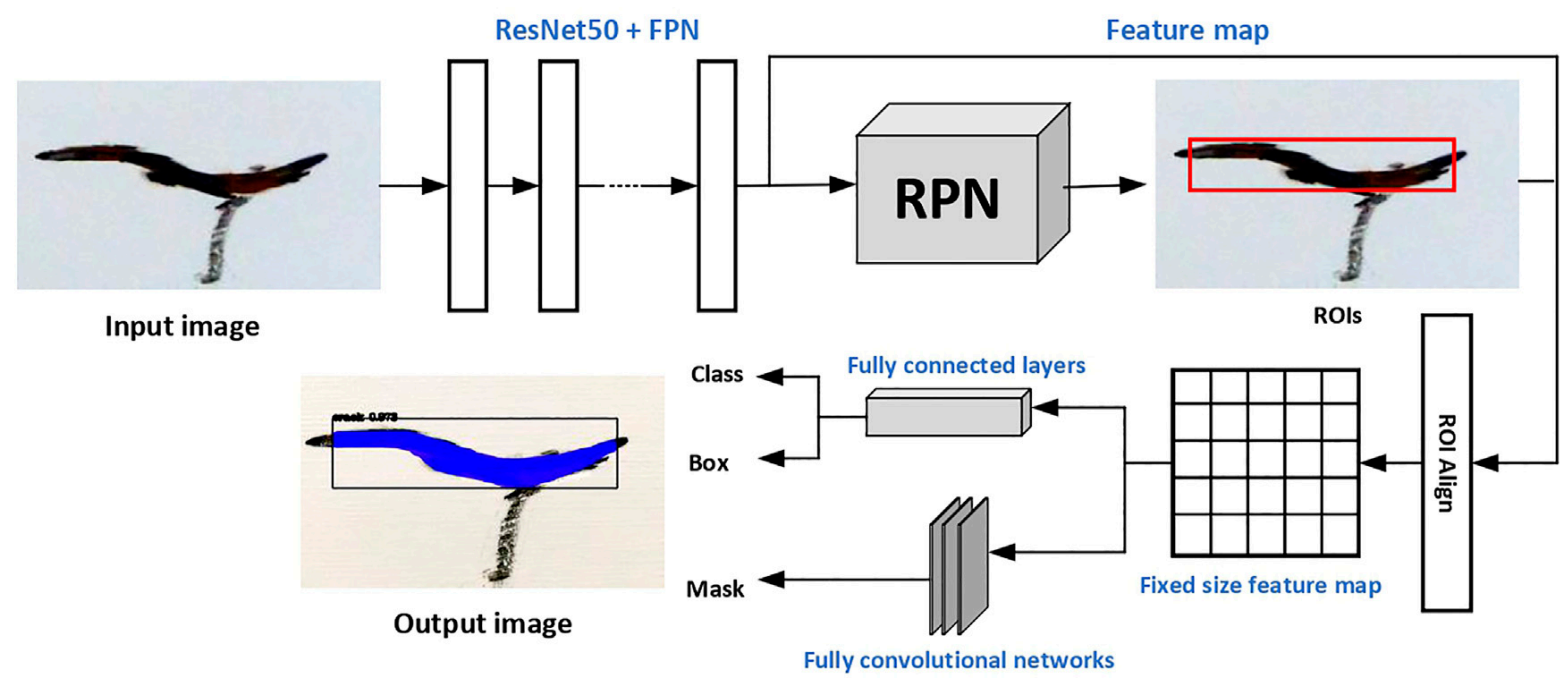
Architecture of the Mask RCNN.

The transfer learning methodology is used in the training of the model. The model is developed based on a pre-trained instance segmentation model Mask R-CNN, which is trained on a large-scale dataset, namely, Microsoft COCO (Lin et al., 2014). As mentioned previously, our annotated dataset is not a large one, while MS COCO contains over 200,000 annotated images. The pre-trained Mask R-CNN model is able to detect 80 different categories of objects, but cracks are not included. Thus, by applying transfer learning, we can train a model on our annotated crack dataset for crack detection, without losing the robustness of the model by fine-tuning the pre-trained one. The model is constructed upon ResNet50 with feature pyramid networks (FPN) as the backbone. The model is flexible and has the ability to be generalized to other instance-level recognition tasks (Zhao et al., 2019), which allows us to fine-tune for crack detection. The multi-task loss function 1 is optimized during the training. The architecture of the adopted model is shown in Figure 4. The crack segmentation task is achieved in two stages: first, the input image is fed to the backbone networks for feature extraction and then region proposal networks (ROI) generate a number of Region of Interest (ROI) candidates in which an optimal one will be obtained through regression. Then the feature map and the chosen ROI are aligned and a fixed-size feature map is obtained; it is sent to two branches, one for the classification and bounding box, while the other generates a mask for pixel-level segmentation.

$$L=L_{\mathrm{class}}+L_{\mathrm{box}}+L_{\mathrm{mask}}$$ (1)

The training stage was carried out with an Intel(R)i7-9850H CPU, 32 GB RAM, and NVIDIA Quadro RTX3000 GPU. The model was built and trained upon the open-source Tensorflow-Keras frame. In the real-time implementation, the model takes an image frame captured by the camera and the target output contains a score, a bounding box, and a binary mask for the detected crack if it exists in the input image.

**FIGURE 5:**
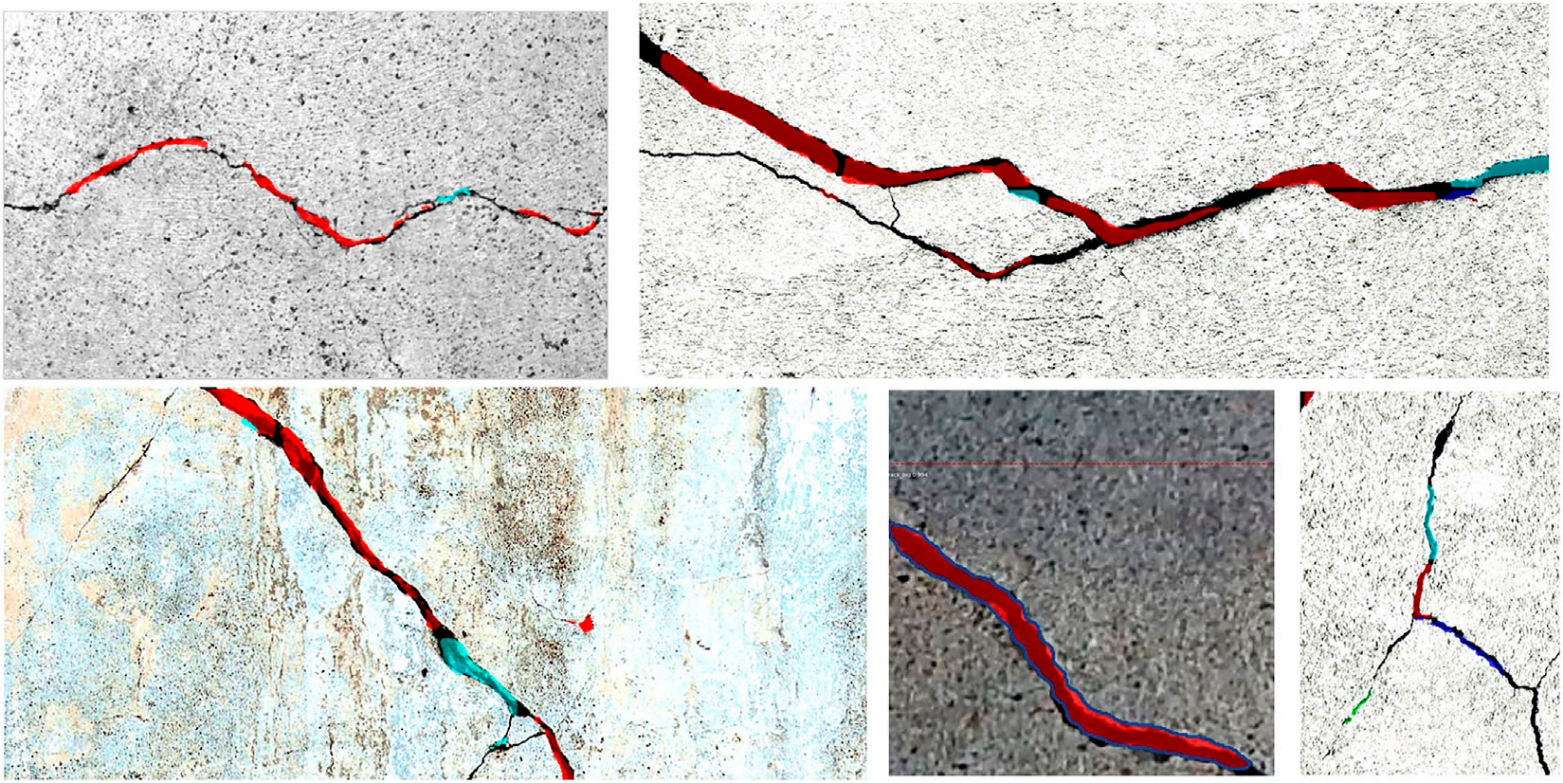
Results of the segmentation model applied on different images of cracks.

**FIGURE 6:**
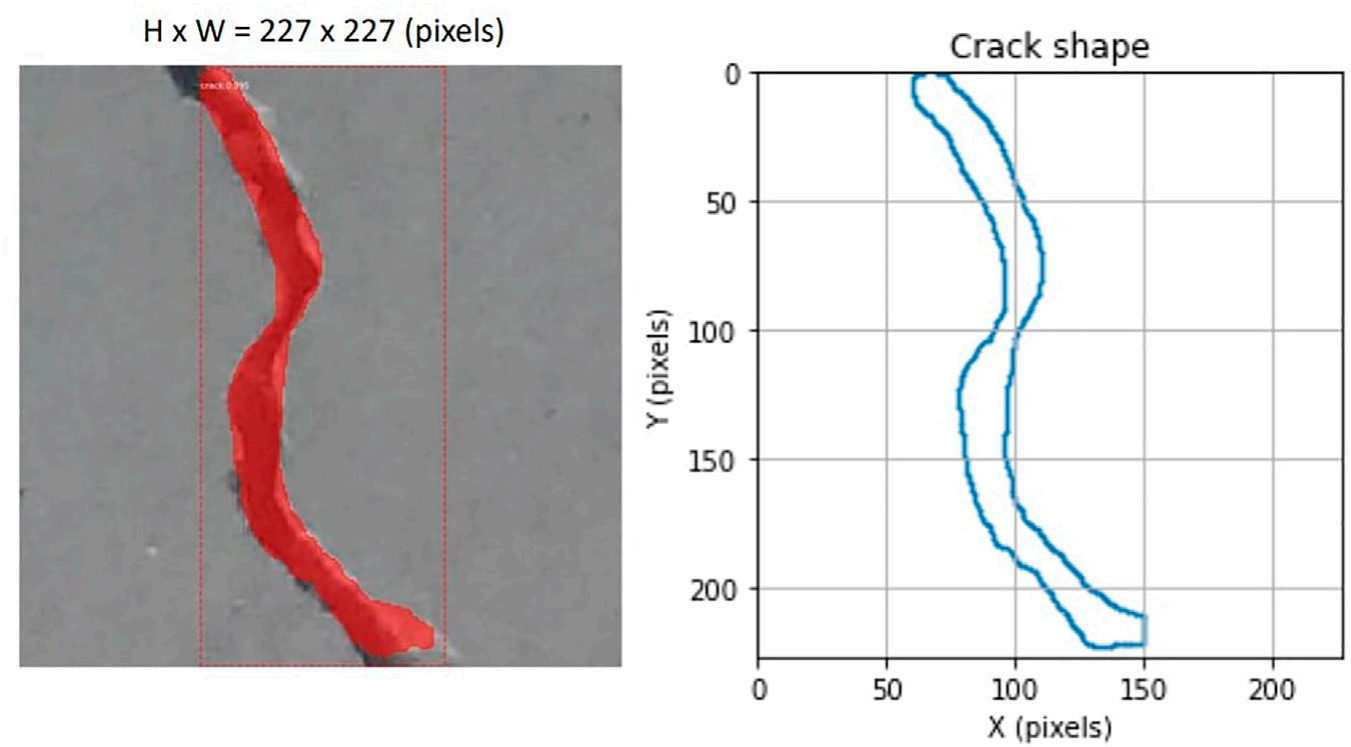
Crack shape reconstruction in the image frame.

As shown in Figure 5, The trained model is tested on some sample images with different sizes that are not contained in the training or validation dataset. Figure 6 shows the bounding boxes and binary masks generated for detected crack instances. The shape of the crack can be reconstructed with its contour in the image coordinate system. The real-time implementation of the model is also tested with the same graphic card on the video stream captured by a USB camera with a resolution of 640 × 480 pixels; it achieves 2 ∼ 4 frames per second (FPS).

### 2.2.2 Crack centerline extraction and localization

To allow the soft arm to access and follow the shape of the detected crack, the localization consists of two steps depicted as follows: centerline extraction and frame transformation.

**FIGURE 7:**
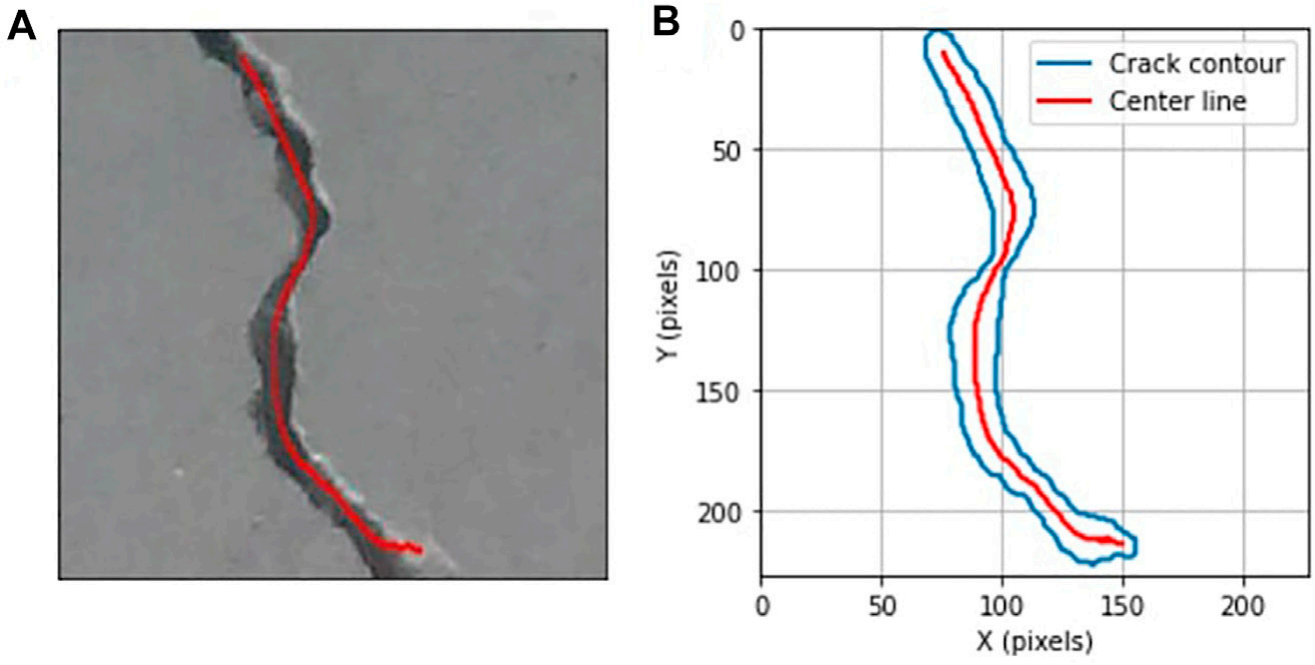
Extraction of the crack centerline in the image coordinate system. **(A)** Crack centerline; **(B)** reconstruction of crack and centerline in the image coordinate system.

First, we extract the centerline of the crack in the image frame. The trajectory to be followed by the robot in the reparation system should represent the structural shape of the detected crack. In the image coordinate system, given *S* is the set of points of the crack region bounded by *C* which is the contour of the crack shape, the centerline of the crack is the set of points *p* ∈ *S* having more than one closest point on the contour *C*. As the output of the detection model contains a binary mask representing the crack, by applying the morphological medial axis transformation algorithm (also referred as skeletonization) on the binary mask, we can obtain the coordinates of the points on the centerline in the image frame. Figure 7 shows an example of crack centerline extraction in the image frame. The medial axis transformation also gives the local widths of the crack at every point on the centerline, expressed in pixels units.

A frame transformation is then applied to the points on the centerline in the image frame obtained from the previous step. For the soft arm to reach the detected crack, it is necessary to locate the centerline in the frame of the soft arm and in the world frame. With a depth camera embedded in the UAV, the distance between the camera and the scanned surface can be obtained.

For the operation of predictive maintenance, the tool path for the end effector of the soft arm robot is identical to the crack centerline. The centerline is expressed as ${\left[\begin{array}{cc} u & v \end{array}\right]}^{T}$ in the pixel frame. In the frame of the scanned surface, the tool path of the end effector of the robot *X*_*ee*_ can be given by

$$X_{ee}=R^{-1}\left(M^{-1}\left(sU\right)-t\right)$$ (2)

where.

• *R* is the rotation matrix of the camera fixed to the UAV.
• *M* is the intrinsic matrix of the camera.
• *t* = [*t*_1_, *t*_2_, *t*_3_] is the translation vector of the camera with respect to the scanned surface frame.
• *s* = *t*_3_ is the depth value, as the UAV stabilizes and is perpendicular to the scanned surface, and it is equal to the distance.
• *U* = [*u v* 0] is the vector of the centerline in the image frame.

**FIGURE 8:**
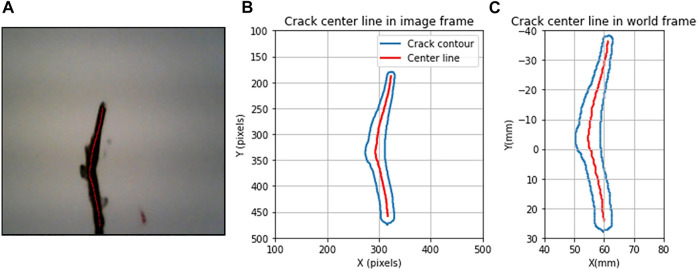
Crack centerline extraction and localization. **(A)** Crack centerline in the raw image; **(B)** reconstruction of crack and centerline in the image coordinate system (pixels); **(C)** reconstruction of crack and centerline in the real dimension (mm).

Figure 8 shows an example of localization of the extracted centerline in real dimension. The unit of the obtained *X*_*ee*_ is in mm and will be sent to the controller of the soft arm robot to execute the maintenance procedure; the robot guides the tip of the nozzle to deposit material for repairing the localized crack in an injection way. The unit of the local widths of the crack is also transformed to mm after the localization step; this allows to classify the crack into microscopic, mesoscopic, and macroscopic according to its size.

The overall processing time from the capture of an image to the calculation of the tool path takes around 0.3 s, allowing a real-time implementation of this visual-based approach.

### 2.3 Model-based inverse kinematic control of the soft arm

#### 2.3.1 Assumptions

The inverse kinematic model (IKM) of the soft arm is developed under the following assumptions:

• The continuum manipulator is considered as a series of N = 8 vertebrae.
• An inter-vertebra is a flexible and non-deformable structure with 3-DoF mobility. It is modeled with 3 UPS–1 UP (3 universal-prismatic-spheric–1 universal-prismatic) joints.
• The manipulator yaw motion is not applicable to the existing mechanical links between the tubes.
• The UAV is considered stable and static in a reference position with contact with the wall, allowing the soft arm to operate autonomously without external disturbances.

**FIGURE 9:**
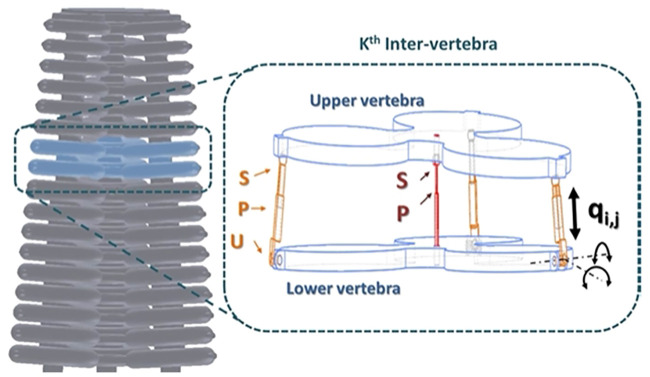
Schematic of an inter-vertebra modeled as a parallel robot with 3 UPS–1 UP.

Based on these assumptions, our soft arm is a series of eight parallel robots composed of 8 vertebrae with 24 DoF in total (Figure 9).

It consists of a lower and upper vertebra connected by three limbs with the same kinematic configuration, and a central leg. The limbs are modeled by a kinematic configuration of type UPS, in which only the prismatic joints are active, allowing to control the position and orientation of the upper vertebra relative to the lower vertebra. *q*_*i*,*j*_ represents the variation of the length of the prismatic joint, where *j* = 1, .., 3 is the index of the active joint and *i* is the index of the frame. The central leg is modeled by a kinematic configuration of type UP located in the center of an inter-vertebra. It is considered a passive joint.

The inverse kinematic equations (IKEs) of an inter-vertebra of the soft continuum manipulator (Figure 9) are obtained by calculating the joint variables *q*_*i*,__*j*=1:3_, corresponding to the pose (position and orientation) of the upper vertebra’s center, relative to the lower vertebra frame. In the case of the considered soft arm, the inter-vertebra is considered as a 3-DoF parallel robot, because of movement constraints related to the passive kinematic chain universal-prismatic (UP). In fact, the rotation with respect to the *z* axis, denoted by *Rot* (*z*, *ϕ*_*i*_), and the translations relative to the *x* and *y* axes denoted by *Trans* (*x*, *X*_*i*_) and *Trans* (*y*, *Y*_*i*_), respectively, are not considered, because it does not exist a movement on these axes. Only the translation along the *z* axis is possible which is denoted by *Trans* (*z*, *Z*_*i*_). Hence, the IKEs can be formulated as follows:

$$q_{i,j}=f\left(Z_{i},\psi_{i},\theta_{i}\right)$$ (3)

where the angles *θ*_*i*_ and *ψ*_*i*_ indicate pitch and roll angles, respectively.

**FIGURE 10:**
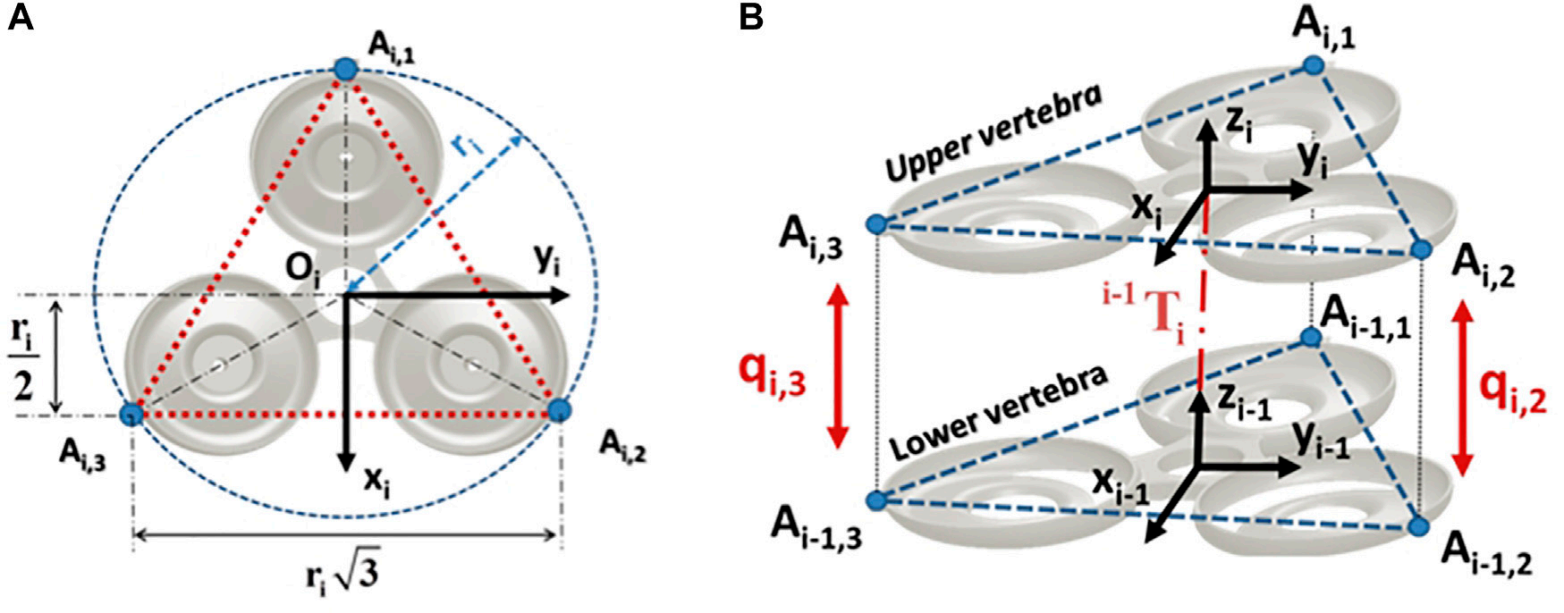
Configuration 3UPS-1UP of the soft arm. **(A)** Configuration of a vertebra. **(B)** Inter-vertebra model.

*A*_*i*,*j*_ represents the connection point between the extensible driving leg *j* = 1, .., 3 and the vertebra *i*, as shown in Figure 10. For each vertebra, the points *A*_*i*,1_ *A*_*i*,2_ *A*_*i*,3_ form an equilateral triangle. The frame *R*_*i*−1_(*O*_*i*−1_, *x*_*i*−1_, *y*_*i*−1_, *z*_*i*−1_) is attached to the lower vertebra of origin *O*_*i*−1_, center of the triangle *A*_*i*−1,1_ *A*_*i*−1,2_ *A*_*i*−1,3_ and the frame *R*_*i*_ (*O*_*i*_, *x*_*i*_, *y*_*i*_, *z*_*i*_) is attached to the upper vertebra of origin *O*_*i*_, located at the center of the equilateral triangle *A*_1,*i*_ *A*_2,*i*_ *A*_3,*i*_. Knowing that the entire shape of the soft arm is conical, it is necessary to find the circumcircle radius *r*_*n*_ of the considered vertebra, where *n* = 1, .., *N* is the number of vertebrae. Let *r*_max_ and *r*_min_, respectively, be the radius of the base and the apex of the backbone, then the radius of each vertebra *r*_*n*_ can be calculated by

$$r_{n}=N_{i}\left(r_{min}-r_{max}\right)+r_{max}$$ (4)

where *N* = 8 is the number of vertebrae. Therefore, the IKE for the *i* ^*th*^ vertebra can be expressed as follows (Lakhal et al., 2015):

$$\begin{array}{c} q_{i,1}^{2}={Z_{i}}^{2}+2r_{i}Z_{i}S\theta_{i}-2r_{i-1}r_{i}C\theta_{k}+{r_{i-1}}^{2}+{r_{i}}^{2}\\ q_{i,2}^{2}={Z_{i}}^{2}+Z_{i}r_{i}\left(\sqrt{3}C\theta_{i}S\psi_{i}-S\theta_{i}\right)-r_{i}r_{i-1}\left(\frac{\sqrt{3}}{2}S\theta_{i}S\psi_{i}+\frac{3}{2}C\psi_{i}+\frac{1}{2}C\theta_{i}\right)+{r_{i-1}}^{2}+{r_{i}}^{2}\\ q_{i,3}^{2}={Z_{i}}^{2}-Z_{k}r_{k}\left(\sqrt{3}C\theta_{k}S\psi_{k}+S\theta_{k}\right)+r_{i}r_{i-1}\left(\frac{\sqrt{3}}{2}S\theta_{i}S\psi_{i}-\frac{3}{2}C\psi_{i}-\frac{1}{2}C\theta_{i}\right)+{r_{i-1}}^{2}+{r_{i}}^{2} \end{array}$$ (5)

The notations *C* and *S* stand for the *cosine* and *sine* functions, respectively.

### 2.3.2 Kinematic tracking control of the soft arm

**FIGURE 11:**
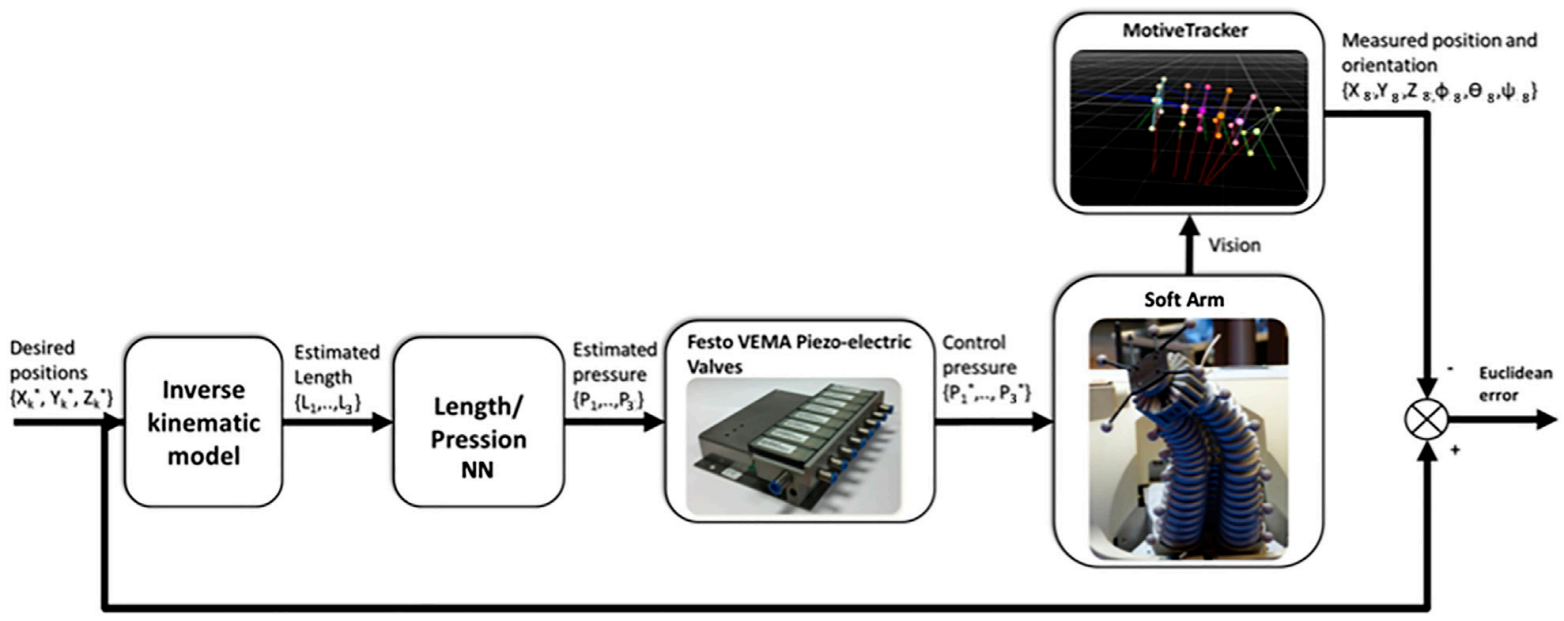
Architecture of the inverse kinematic model-based control validation.

The inverse kinematics tracking control proceeds as shown in Figure 11. The desired posture of the soft arm is generated by applying a set of the desired pressures to the three bending tubes. The desired positions of the soft arm tip is applied to IKM, and the predicted lengths generated by the IKM are used as input to the length-pressure converter, approximated by a multi-layer perceptron neural network (MLPNN) (Melingui et al., 2017) as shown in Table 1.

**TABLE 1:** Results achieved by each neural network model on the test samples.

Neural network topologies	Neurons	MSE
MLP, pressures section 1 (2 layers)	11	4.3242.10^–5^
MLP, pressures section 2 (2 layers)	11	3.7519.10^–5^

**FIGURE 12:**
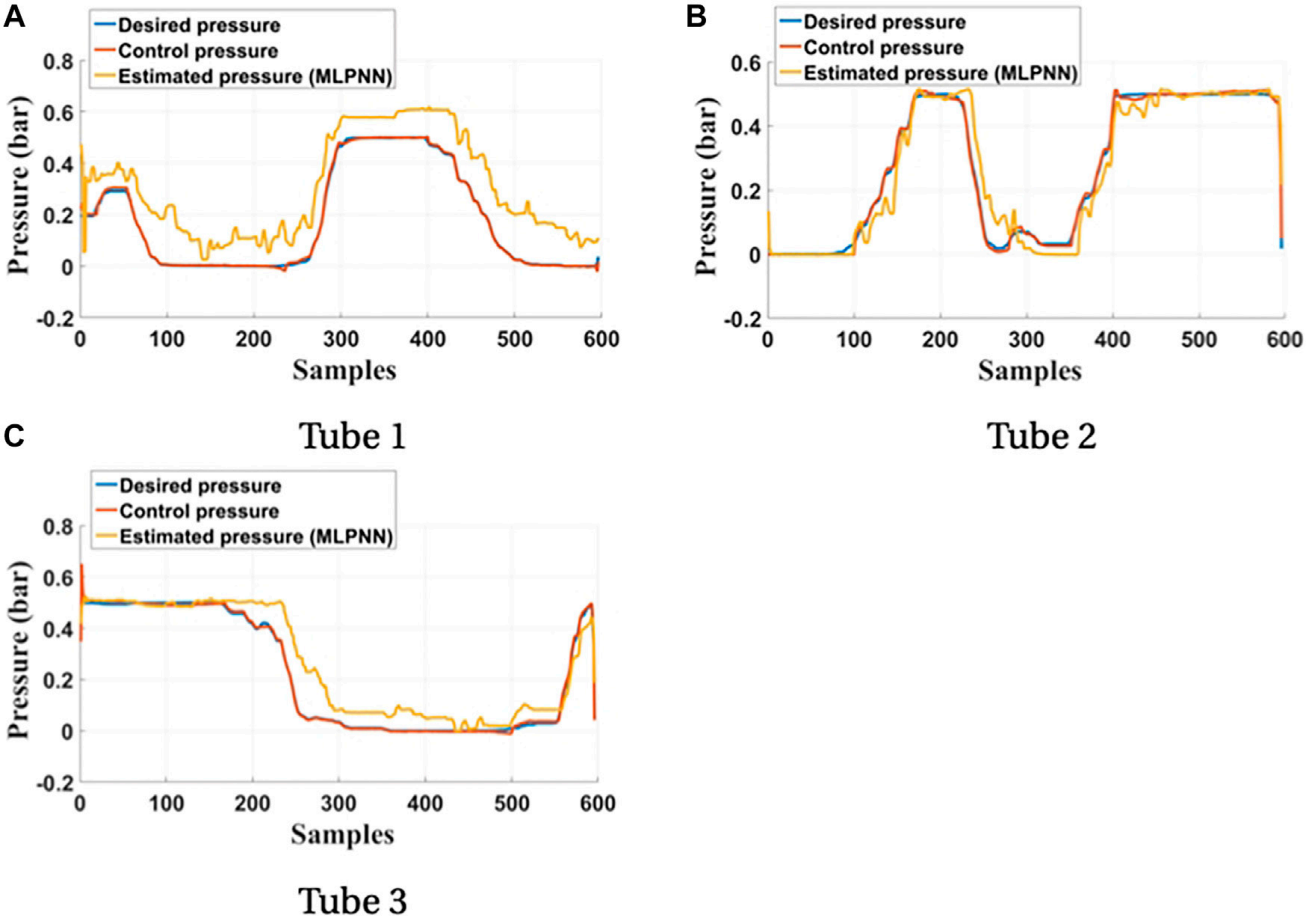
Comparison between the desired pressures and the predicted pressures of the three bending tubes. **(A)** Tube 1. **(B)** Tube 2. **(C)** Tube 3.

The pressures generated by the length-pressure converter are applied to the soft arm by means of the internal proportional–integral–derivative (PID) controllers of the Festo VEMA piezo-electric valves. The position predicted by the Optitrack system is compared with the desired positions shown in Figure 12.

## 3 Results

This section presents the experiment results of the applied predictive maintenance. It focuses on the validation of the developed kinematic models. Different cracks are considered on a tile plate sample to validate the algorithms of detection, localization, and repair.

### 3.1 Real-time implementation

**FIGURE 13:**
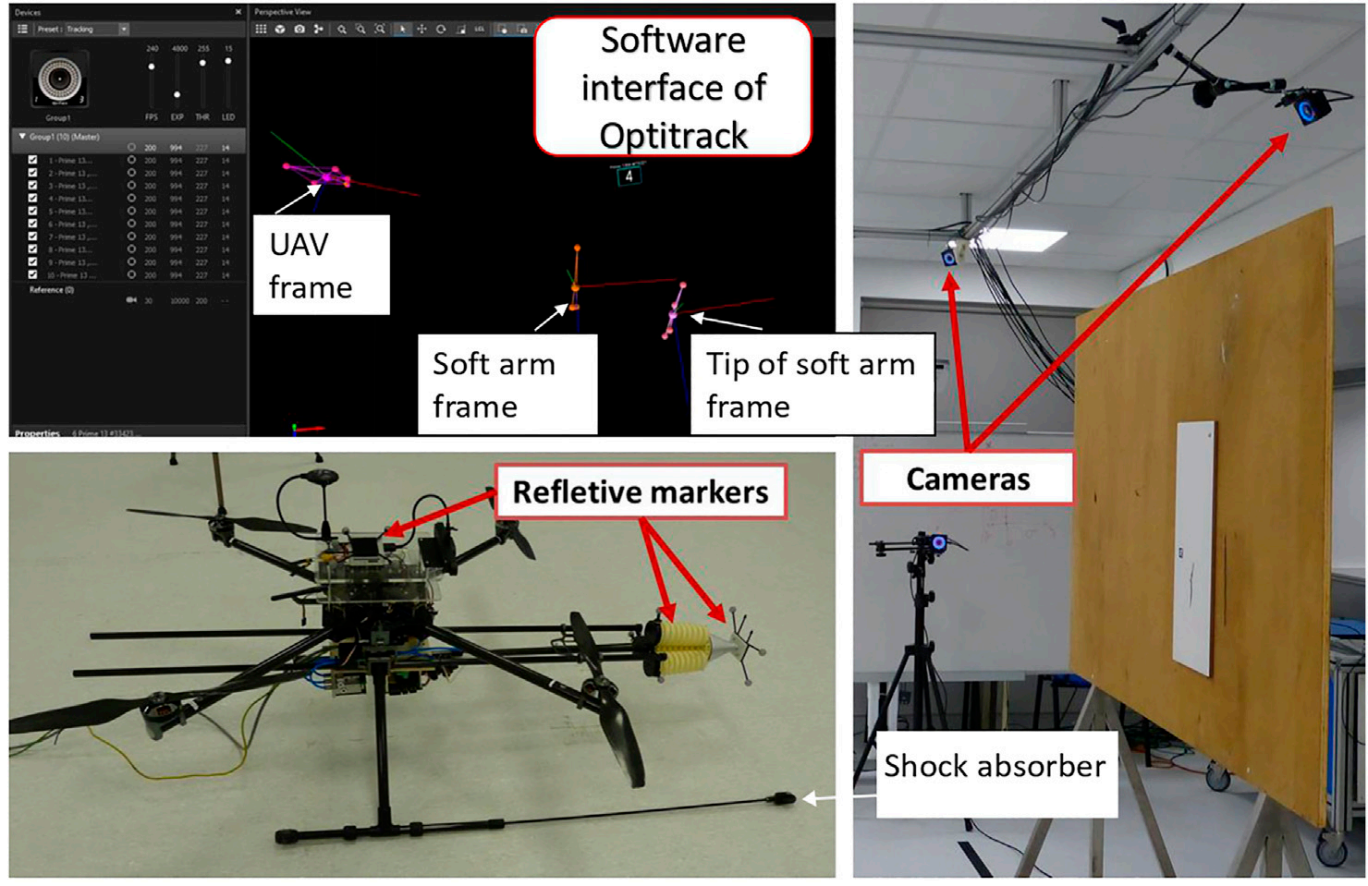
Stereo-vision system for trajectory tracking.

The tracking process is shown in Figure 13. The system is based on the Optitrack motion capture system and consists of a set of 10 infrared cameras, as described in the previous paragraph. After calibration, a precision of 0.3 *mm* for measuring the 3-D displacements of the UAV and the tip of the arm is obtained. The reflection markers placed at the base of the arm are used to obtain the coordinates of the base relative to the UAV.

### 3.2 Experimental results

**FIGURE 14:**
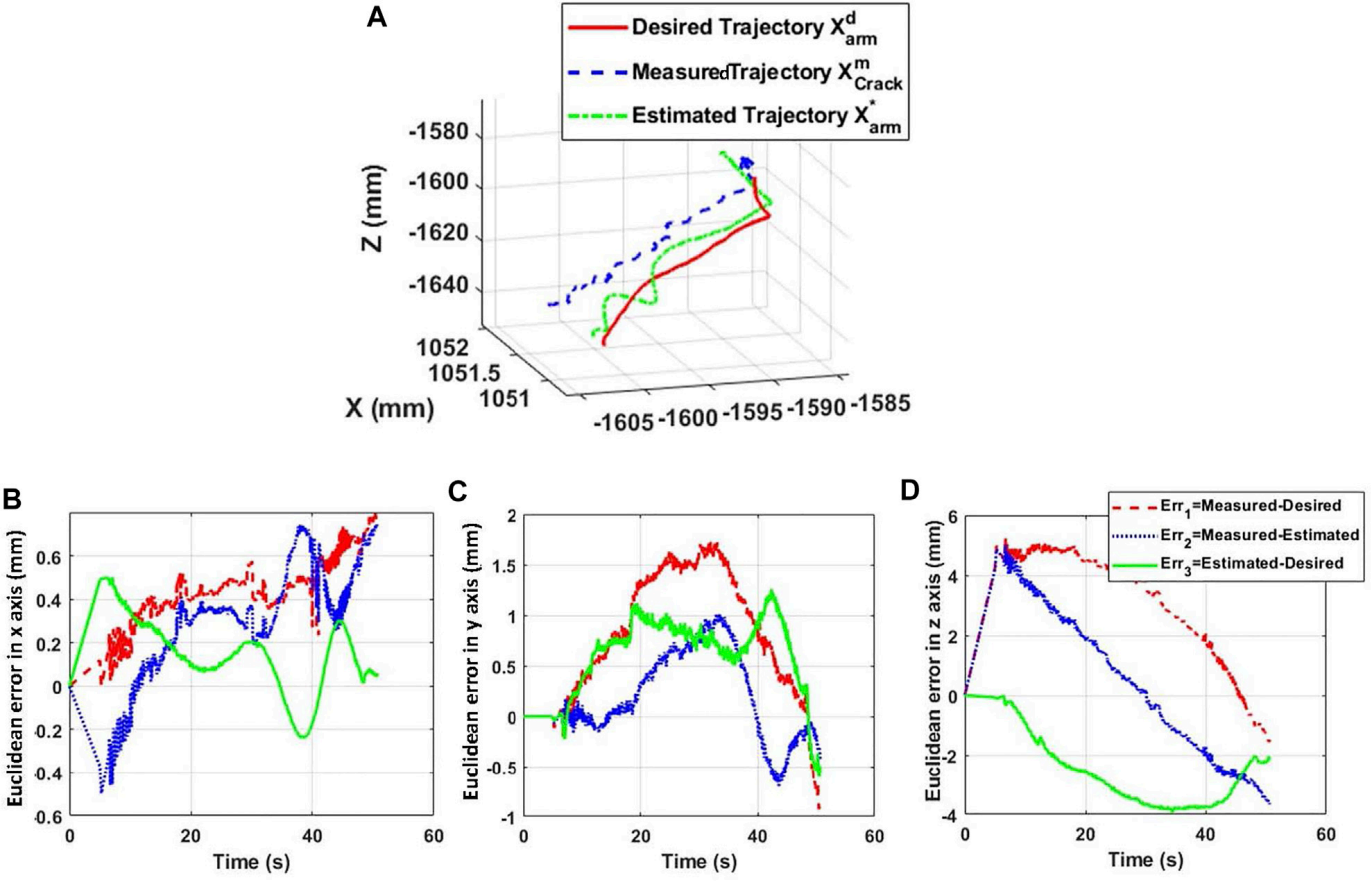
Trajectory of the tip of the arm during the reparation process of crack 1. **(A)** 3D position tracking of crack 1. **(B)** *X*-axis error. **(C)** *Y*-axis error. **(D)** *Z*-axis error.

**FIGURE 15:**
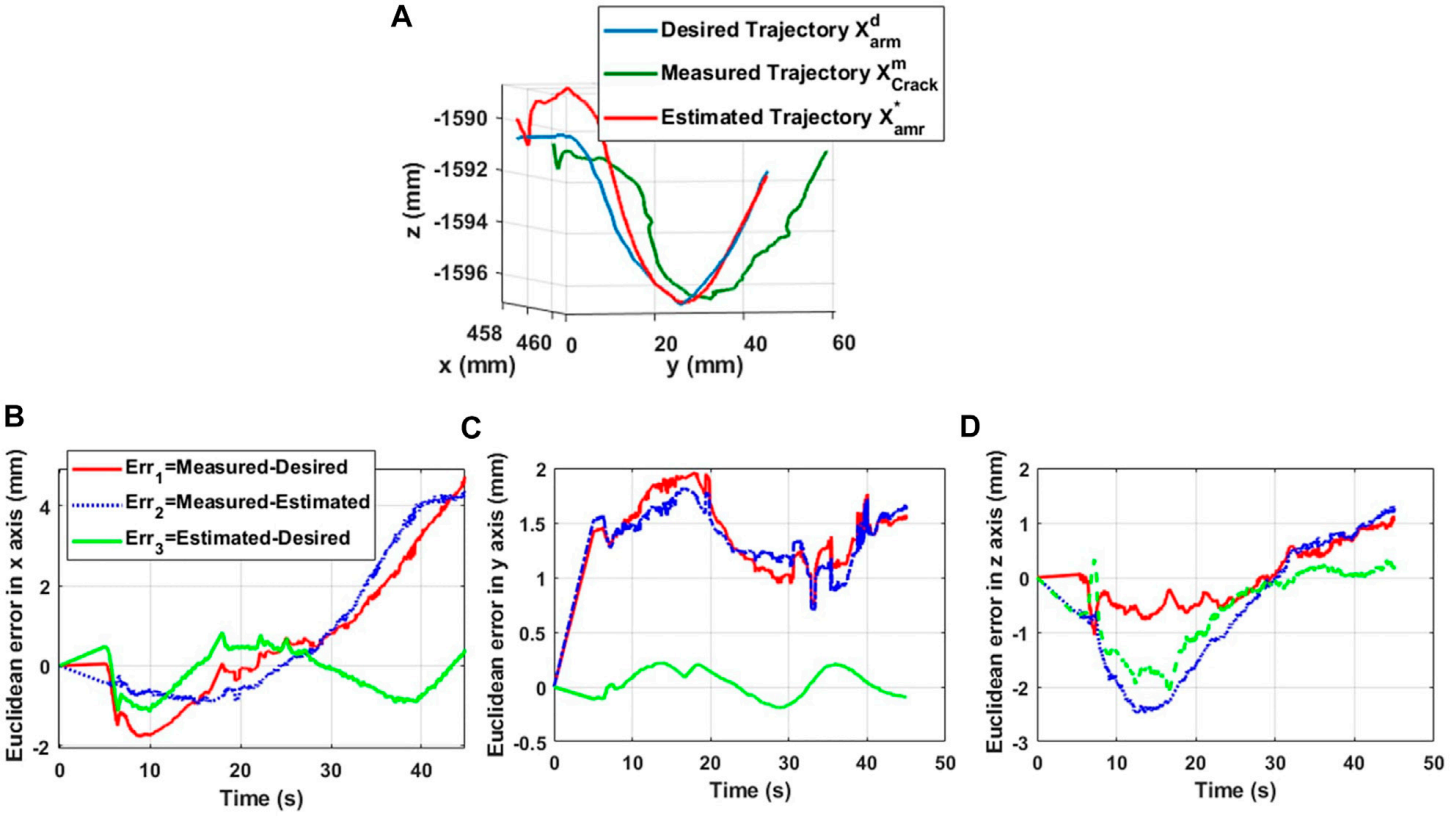
Trajectory of the tip of the arm during the reparation process of crack 2. **(A)** 3D position tracking of crack 2. **(B)** *X*-axis error. **(C)** *Y*-axis error. **(D)** *Z*-axis error.

Figure 14A, Figure15A show the trajectories of the tip of the arm (nozzle) during the deposit of putty materials on crack 1 (vertical shape) and crack 2 (horizontal shape), respectively. The desired trajectories represent the center-lines of the reconstructed shapes of the cracks after a crack detection and classification technique. The measured trajectory is issued from the Optitrack system and the estimated trajectory is generated from the inverse kinematic model of the soft arm. It is noticed from these experimental tests that the estimated kinematic model reconstructs globally the desired trajectory, while the measured trajectory based on the Optitrack vision capture is perhaps sensitive to the micro-motion of the UAV in its stationary positioning. Figures 14B, C represent the Euclidean errors along *x*, *y,* and *z* axes for crack 1, whereas, Figures 15B , 15c, and 15d represent the Euclidean errors along *x*, *y,* and *z* axes, respectively, for crack 2. It is noticed that the errors are less than 5 mm. However, the error in z can be relatively higher than the others and can be explained by the gravity effect on the soft arm.

Euclidean errors in Figure 14 and Figure 15 conclude that the proposed kinematics model is able to predict the position of the tip of the nozzle with position errors less than 5 mm, where the nozzle diameter is about 5 mm.

**FIGURE 16:**
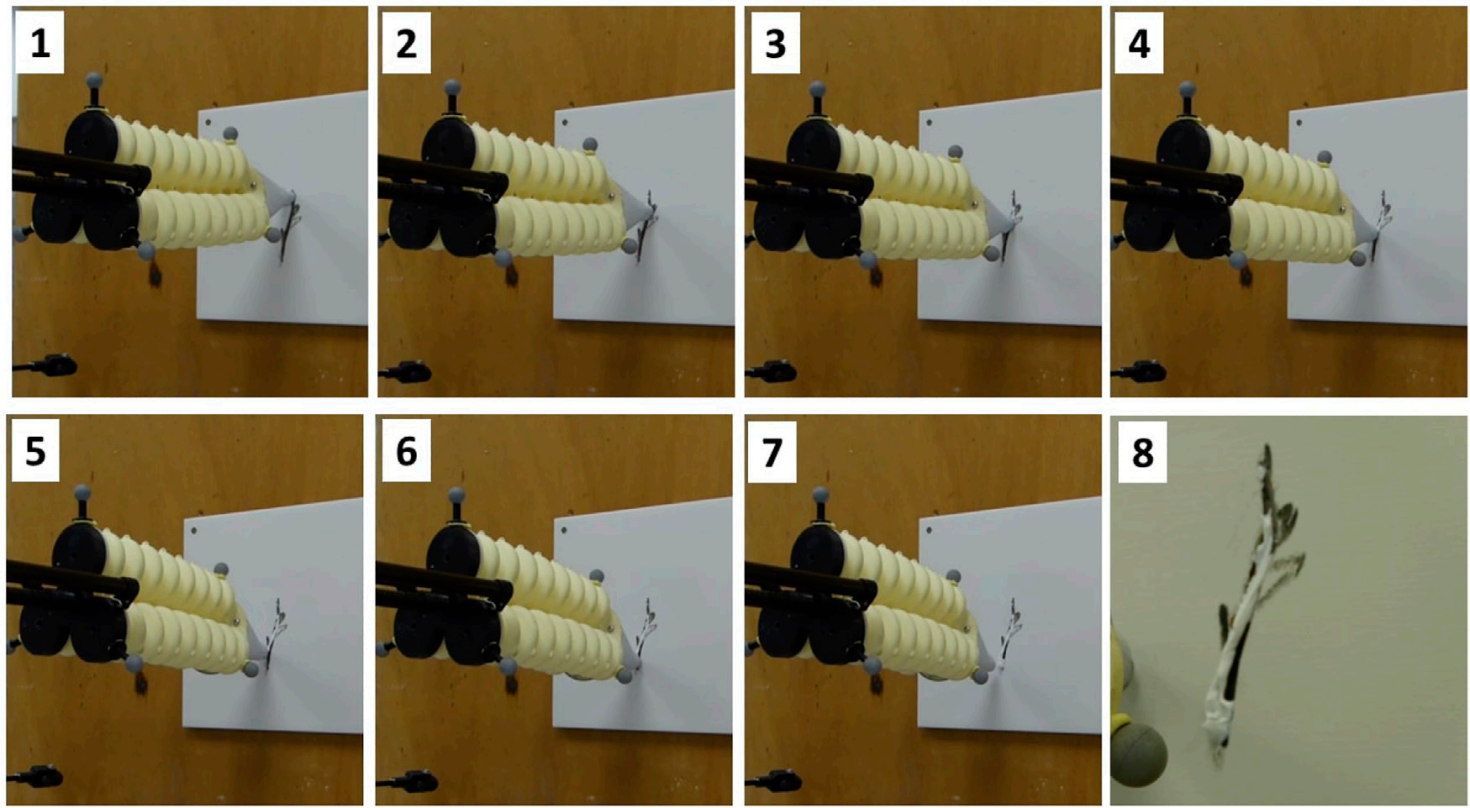
Repair steps for crack 1.

In Figure 16, a sequential representation of the deposit material during the deposit of a putty material is shown. The accuracy of tracking tasks by the system is shown in the video (Lakhal et al., 2021), for online target tracking.

Finally, based on the results shown in Figure 16 and the Euclidean errors in Figure 14, the performance of the microscopic crack detection and localization using the deep learning technique is demonstrated.

## 4 Discussion

An artificial intelligence-based method using deep learning techniques has been developed to detect and repair cracks automatically. After applying the automatic detection algorithm to the wall surface, the classification of the cracks is obtained by dividing them according to their sizes into microscopic (less than 5 mm width), mesoscopic (in between 5 and 10 mm width), and macroscopic (more than 10 mm width). In this work, we focused on the microscopic cracks, where the preparation of the surface is not required and only the repair is needed. Then, the localization of the centerline of the crack shape is determined. Using the current position of the UAV, the ultrasonic sensor and the camera embedded in the robot give the measures for planning the trajectory to be followed by the tip of the soft continuum arm. The model-based control of the position of the arm tip is applied along the crack with various disturbances such as not-zero stability of the UAV but also the contact of the soft arm during the deposit of the material. This is because errors are corrected by the soft arm, which has faster and more accurate stability than the millimeter-scale drone. The stability of a UAV in flight is slightly more complex, especially for millimeter-scale movements. In our case study, the UAV is in a stationary position throughout the repair, which means that the sum of the opposing forces is zero, and therefore, the displacement of the system’s center of gravity is small due to the ground effect. In addition, when the soft arm comes into contact with the wall, it unloads to maintain a constant force when depositing the material, but also to avoid large disturbances to the movement of the drone that could involve a crash. Indeed, the combination of the soft arm capabilities and the flying mobile platform offers great cooperation to perform an operational task. The soft arm can reach complex positions of the crack, while the UAV can guide and load the arm with different postures. In view of the results obtained, the proposed concept achieves promising performances in terms of precision and robustness for automatic detection and repairs in predictive construction maintenance. The accuracy is in the millimeter range. The trajectory tracking is well-respected despite the total instability of the drone. However, as we can see from the curves, the intervention time is about 60 s for a crack of about 80 *mm* in size. In fact, for precise control and optimal material deposition quality, it is preferable to work in quasi-static kinematics. If one wishes to increase the speed of loading and unloading of the pressure of the flexible arm, it is necessary to consider using other types of sensors than piezos. In Figure 16, the results are relatively satisfactory in terms of the quality of the material deposit. Indeed, a more or less identical contact force is obtained along the whole trajectory. Nevertheless, we have determined the gain by assuming that the forces are exerted only on the *z* axis. However, curved trajectories require larger deformations of the flexible arm, which implies that the contact force is not only on the *z*-axis but on all three axes.

## 5 Conclusion

This study deals with an integrated robotic concept design for automatic crack detection and repair. This concept is composed of a stationary UAV and a soft continuum arm. The latter is used to guide the putty material but also to reach complex shapes of microscopic cracks in flat and oblique surfaces of wall surfaces. The concept integrates the tasks of detection and localization of the crack shapes using deep learning techniques. The repair task is summarized by a continuous deposit of the material to fill the cracks. The stationary UAV interacts with the wall surface using wheeled contact, while the soft arm is not directly in contact with the external surface. The methodology of the inverse kinematic control of the soft arm has been deployed. A neural network-based predictive model allows for estimating the relationship between the bending tube’s length and the input pressure. The experimental results show the performance of the kinematic control as a function of material deposition accuracy. In future work, the objective is to implement a joint control on the robot position and the material deposition flow rate, as a function of the width and the deep of the crack, to improve the quality of the deposit.

## Data Availability

The raw data supporting the conclusion of this article will be made available by the authors, without undue reservation.

## References

[B1] Aggelis D. Kordatos E. Strantza M. Soulioti D. Matikas T. (2011). Ndt approach for characterization of subsurface cracks in concrete. Constr. Build. Mater. 25, 3089–3097. 10.1016/j.conbuildmat.2010.12.045

[B2] Albishi A. M. Boybay M. S. Ramahi O. M. (2012). Complementary split-ring resonator for crack detection in metallic surfaces. IEEE Microw. Wirel. Compon. Lett. 22, 330–332. 10.1109/LMWC.2012.2197384

[B3] Anantharaman R. Velazquez M. Lee Y. (2018). “Utilizing mask r-cnn for detection and segmentation of oral diseases,” in 2018 IEEE international conference on bioinformatics and biomedicine (BIBM) (IEEE).

[B4] Chen I.-M. Asadi E. Nie J. Yan R.-J. Law W. C. Kayacan E. (2016). “Innovations in infrastructure service robots,” in ROMANSY 21-robot design, dynamics and control (Springer), 3–16.

[B5] Daachi B. Madani T. Benallegue A. (2012). Adaptive neural controller for redundant robot manipulators and collision avoidance with mobile obstacles. Neurocomputing 79, 50–60. 10.1016/j.neucom.2011.10.001

[B6] De Paz J. P. Z. Castañeda E. C. Castro X. Y. S. Jiménez S. M. R. (2013). “Crack detection by a climbing robot using image analysis,” in CONIELECOMP 2013, 23rd international conference on electronics, communications and computing, 87–91. 10.1109/CONIELECOMP.2013.6525765

[B7] Dorafshan S. Maguire M. (2018). Bridge inspection: Human performance, unmanned aerial systems and automation. J. Civ. Struct. Health Monit. 8, 443–476. 10.1007/s13349-018-0285-4

[B8] Dorafshan S. Maguire M. Hoffer N. V. Coopmans C. (2017). “Challenges in bridge inspection using small unmanned aerial systems: Results and lessons learned,” in 2017 international conference on unmanned aircraft systems (ICUAS) (IEEE), 1722–1730.

[B9] Dorafshan S. Thomas R. J. Coopmans C. Maguire M. (2018a). “Deep learning neural networks for suas-assisted structural inspections: Feasibility and application,” in 2018 international conference on unmanned aircraft systems (ICUAS) (IEEE), 874–882.

[B10] Dorafshan S. Thomas R. J. Maguire M. (2018b). Comparison of deep convolutional neural networks and edge detectors for image-based crack detection in concrete. Constr. Build. Mater. 186, 1031–1045. 10.1016/j.conbuildmat.2018.08.011

[B11] Dorafshan S. Thomas R. J. Maguire M. (2018c). Sdnet2018: An annotated image dataset for non-contact concrete crack detection using deep convolutional neural networks. Data brief 21, 1664–1668. 10.1016/j.dib.2018.11.015 30505897 PMC6247444

[B12] Escande C. Chettibi T. Merzouki R. Coelen V. Pathak P. M. (2015). Kinematic calibration of a multisection bionic manipulator. Ieee. ASME. Trans. Mechatron. 20, 663–674. 10.1109/TMECH.2014.2313741

[B13] Feng W. Hu Z. Wu W. Yan J. Ouyang W. (2019). Multi-object tracking with multiple cues and switcher-aware classification. arXiv preprint arXiv:1901.06129.

[B14] Flah M. Suleiman A. R. Nehdi M. L. (2020). Classification and quantification of cracks in concrete structures using deep learning image-based techniques. Cem. Concr. Compos. 114, 103781. 10.1016/j.cemconcomp.2020.103781

[B15] Hameed K. Chai D. Rassau A. (2022). Score-based mask edge improvement of mask-rcnn for segmentation of fruit and vegetables. Expert Syst. Appl. 190, 116205. 10.1016/j.eswa.2021.116205

[B16] He K. Gkioxari G. Dollár P. Girshick R. (2017). “Mask r-cnn,” in Proceedings of the IEEE international conference on computer vision, 2961–2969.

[B17] Jia W. Tian Y. Luo R. Zhang Z. Lian J. Zheng Y. (2020). Detection and segmentation of overlapped fruits based on optimized mask r-cnn application in apple harvesting robot. Comput. Electron. Agric. 172, 105380. 10.1016/j.compag.2020.105380

[B18] Jiang H. Wang Z. Liu X. Chen X. Jin Y. You X. (2017). “A two-level approach for solving the inverse kinematics of an extensible soft arm considering viscoelastic behavior,” in 2017 IEEE international conference on robotics and automation (ICRA), 6127–6133. 10.1109/ICRA.2017.7989727

[B19] Karlik B. Aydin S. (2000). An improved approach to the solution of inverse kinematics problems for robot manipulators. Eng. Appl. Artif. Intell. 13, 159–164. 10.1016/S0952-1976(99)00050-0

[B20] Krizhevsky A. Sutskever I. Hinton G. E. (2017). Imagenet classification with deep convolutional neural networks. Commun. ACM 60, 84–90. 10.1145/3065386

[B21] Kucuksubasi F. Sorguc A. (2018). Transfer learning-based crack detection by autonomous uavs. CoRR abs/1807, 11785. 10.48550/arXiv.1807.11785

[B22] Lakhal O. Kahouadji M. Yang X. (2021). Detection and crack reparation with uav. Available at: https://drive.google.com/file/d/1AFDw5UIIs0tjcsSDvQApTC1wLDpGQSre/view?usp=sharing.

[B23] Lakhal O. Melingui A. Merzouki R. (2015). Hybrid approach for modeling and solving of kinematics of a compact bionic handling assistant manipulator. Ieee. ASME. Trans. Mechatron. 21, 1326–1335. 10.1109/tmech.2015.2490180

[B24] Lau K.-T. (2003). Fibre-optic sensors and smart composites for concrete applications. Mag. Concr. Res. 55, 19–34. 10.1680/macr.2003.55.1.19

[B25] Li Y. Chen X. Zhu Z. Xie L. Huang G. Du D. (2019). “Attention-guided unified network for panoptic segmentation,” in Proceedings of the IEEE conference on computer vision and pattern recognition, 7026–7035.

[B26] Lin T.-Y. Maire M. Belongie S. Hays J. Perona P. Ramanan D. (2014). “Microsoft coco: Common objects in context,” in European conference on computer vision (Springer), 740–755.

[B27] Maguire M. Dorafshan S. Thomas R. J. (2018). Sdnet2018: A concrete crack image dataset for machine learning applications. 10.1016/j.dib.2018.11.015 PMC6247444 30505897

[B28] Melingui A. Ahanda J. J.-B. M. Lakhal O. Mbede J. B. Merzouki R. (2017). Adaptive algorithms for performance improvement of a class of continuum manipulators. IEEE Trans. Syst. Man. Cybern. Syst. 48, 1531–1541. 10.1109/tsmc.2017.2678605

[B29] Melingui A. Escande C. Benoudjit N. Merzouki R. Mbede J. (2014). 19th IFAC World Congress, 47, 9353–9358. 10.3182/20140824-6-ZA-1003.01758 Qualitative approach for forward kinematic modeling of a compact bionic handling assistant trunk IFAC Proc. Vol.

[B30] Melingui A. Lakhal O. Daachi B. Mbede J. B. Merzouki R. (2015). Adaptive neural network control of a compact bionic handling arm. Ieee. ASME. Trans. Mechatron. 20, 2862–2875. 10.1109/TMECH.2015.2396114

[B31] Mohan A. Poobal S. (2018). Crack detection using image processing: A critical review and analysis. Alexandria Eng. J. 57, 787–798. 10.1016/j.aej.2017.01.020

[B32] Oliveira H. Correia P. L. (2013). Automatic road crack detection and characterization. IEEE Trans. Intell. Transp. Syst. 14, 155–168. 10.1109/TITS.2012.2208630

[B33] Özgenel Ç. F. Gönenç Sorguç A. “Performance Comparison of Pretrained Convolutional Neural Networks on Crack Detection in Buildings,” ISARC 2018 Berlin.

[B34] Palermo F. Konstantinova J. Althoefer K. Poslad S. Farkhatdinov I. (2020). “Implementing tactile and proximity sensing for crack detection,” in 2020 IEEE international conference on robotics and automation (ICRA) (IEEE), 632–637.

[B35] Park S. E. Eem S.-H. Jeon H. (2020). Concrete crack detection and quantification using deep learning and structured light. Constr. Build. Mater. 252, 119096. 10.1016/j.conbuildmat.2020.119096

[B36] Perez H. Tah J. H. Mosavi A. (2019). Deep learning for detecting building defects using convolutional neural networks. Sensors 19, 3556. 10.3390/s19163556 PMC6720984 31443244

[B37] Phung M. D. Hoang V. T. Dinh T. H. Ha Q. (2017). Automatic crack detection in built infrastructure using unmanned aerial vehicles. arXiv preprint arXiv:1707.09715.

[B38] Prasanna P. Dana K. J. Gucunski N. Basily B. B. La H. M. Lim R. S. (2016). Automated crack detection on concrete bridges. IEEE Trans. Autom. Sci. Eng. 13, 591–599. 10.1109/tase.2014.2354314

[B39] Reinhart R. F. Shareef Z. Steil J. J. (2017). Hybrid analytical and data-driven modeling for feed-forward robot control †. Sensors 17, 311. 10.3390/s17020311 PMC5336126 28208697

[B40] Shi Y. Cui L. Qi Z. Meng F. Chen Z. (2016). Automatic road crack detection using random structured forests. IEEE Trans. Intell. Transp. Syst. 17, 3434–3445. 10.1109/tits.2016.2552248

[B41] Silva W. R. L. d. Lucena D. S. d. (2018). Concrete cracks detection based on deep learning image classification. Multidiscip. Digit. Publ. Inst. Proc. 2, 489.

[B42] Tsai Y. J. Kaul V. Yezzi A. (2013). Automating the crack map detection process for machine operated crack sealer. Automation Constr. 31, 10–18. 10.1016/j.autcon.2012.11.033

[B43] Yan R.-J. Kayacan E. Chen I.-M. Tiong L. K. (2017). “A novel building post-construction quality assessment robot: Design and prototyping,” in Intelligent robots and systems (IROS), 2017 IEEE/RSJ international conference on (IEEE), 6020–6023.

[B44] Yao G. Wei F. Yang Y. Sun Y. (2019). Deep-learning-based bughole detection for concrete surface image. Adv. Civ. Eng., 1–12. 10.1155/2019/8582963

[B45] Zhang W. Zhang Z. Qi D. Liu Y. (2014). Automatic crack detection and classification method for subway tunnel safety monitoring. Sensors 14, 19307–19328. 10.3390/s141019307 25325337 PMC4239952

[B46] Zhao Z.-Q. Zheng P. Xu S.-t. Wu X. (2019). Object detection with deep learning: A review. IEEE Trans. Neural Netw. Learn. Syst. 30, 3212–3232. 10.1109/tnnls.2018.2876865 30703038

[B47] Zhu G. Fan Z. Chen W. You Y. Huang S. Liang W. (2019). “Design and implementation of a manipulator system for roadway crack sealing,” in 2019 IEEE 9th annual international conference on CYBER technology in automation, control, and intelligent systems (CYBER), 1327–1331. 10.1109/CYBER46603.2019.9066587

